# Ambient PM exposure and DNA methylation in tumor suppressor genes: a cross-sectional study

**DOI:** 10.1186/1743-8977-8-25

**Published:** 2011-08-30

**Authors:** Lifang Hou, Xiao Zhang, Letizia Tarantini, Francesco Nordio, Matteo Bonzini, Laura Angelici, Barbara Marinelli, Giovanna Rizzo, Laura Cantone, Pietro Apostoli, Pier Alberto Bertazzi, Andrea Baccarelli

**Affiliations:** 1 Department of Preventive Medicine, Feinberg School of Medicine, Northwestern University, 680 N. Lakeshore Drive, Chicago, 60611, USA; 2 Robert H. Lurie Comprehensive Cancer Center Feinberg School of Medicine, Northwestern University, 303 E Superior Street, Chicago, 60611, USA; 3 Department of Preventive Medicine and Department of Environmental and Occupational Health, University of Milan and IRCCS Maggiore Hospital, Mangiagalli and Regina Elena Foundation, Via Pace 9, Milan, 20122, Italy; 4 Department of Clinical and Biological Sciences, University of Insubria, Viale Borri 57, Varese, 21100 Italy; 5 Department of Experimental and Applied Medicine, Occupational Medicine and Industrial Hygiene, University of Brescia, P.le Spedali Civili 1, Brescia, 25123, Italy; 6 Laboratory of Environmental Epigenetics, Department of Environmental Health, Harvard School of Public Health, 665 Huntington Ave, Boston, 02115, USA; 7 Department of Clinical Medicine, Nephrology and Health Science, University of Parma Medical School, Via Gramsci 14, Parma, 43126, Italy

## Abstract

Exposure to ambient air particles matter (PM) has been associated with increased risk of lung cancer. Aberrant tumor suppressor gene promoter methylation has emerged as a promising biomarker for cancers, including lung cancer. Whether exposure to PM is associated with peripheral blood leukocyte (PBL) DNA methylation in tumor suppressor genes has not been evaluated. In 63 male healthy steel workers with well-characterized exposure to metal-rich particles nearby Brescia, Italy, we evaluated whether exposure to PM and metal components was associated with PBL DNA methylation in 4 tumor suppressor genes (i.e., APC, p16, p53 and RASSF1A). Blood samples were obtained on the 1^st ^(baseline) and 4^th ^day (post-exposure) of the same work week and DNA methylation was measured using pyrosequencing. A linear mixed model was used to examine the correlations of the exposure with promoter methylation levels. Mean promoter DNA methylation levels of APC or p16 were significantly higher in post-exposure samples compared to that in baseline samples (p-values = 0.005 for APC, and p-value = 0.006 for p16). By contrast, the mean levels of p53 or RASSF1A promoter methylation was decreased in post-exposure samples compared to that in baseline samples (p-value = 0.015 for p53; and p-value < 0.001 for RASSF1A). In post-exposure samples, APC methylation was positively associated with PM_10 _(β = 0.27, 95% CI: 0.13-0.40), and PM_1 _(β = 0.23, 95% CI: 0.09-0.38). In summary, ambient PM exposure was associated with PBL DNA methylation levels of tumor suppressor genes of APC, p16, p53 and RASSF1A, suggesting that such methylation alterations may reflect processes related to PM-induced lung carcinogenesis.

## Introduction

Ambient and occupational exposure to particular matter (PM) has been associated with increased risk of lung cancer [1,2]. Although the carcinogenic potential of several toxic metals in PM was well-recognized, the molecular mechanisms underlying their association with cancer risks remain poorly understood. Experimental and epidemiologic studies suggest that PM mass and metal components may induce critical carcinogenesis-related biological changes, including oxidative stress, immune deficiency, and chronic inflammation, which have recently been shown to alter gene expression via DNA methylation mechanism [3].

Growing evidence indicates that epigenetic dysregulation of gene expression plays a primary role in cancer etiology [4,5]. Methylation of 5'CpG islands in promoter region has emerged as one of the most important epigenetic mechanisms in the development of human cancers [6]. Aberrant promoter methylation of a series of tumor suppressor genes has been detected in blood leukocyte DNA from lung cancer patients [7] and healthy subjects exposed to carcinogens [8,9]. In our previous studies, we have reported blood leukocyte global hypomethylation in subjects exposed to PM and metal components [10-12] and benzene [13]. Global hypomethylation is frequently observed in cancer tissues, including lung cancer [14,15] and blood leukocytes of cancer patients [16] and it often co-exists with gene-specific methylation alterations [17]. However, whether ambient PM and its metal components can induce DNA methylation alterations in tumor suppressor genes, which may be involved in air pollution-related lung carcinogenesis, has not been examined.

DNA methylation is mitotically stable and can be propagated through cell division from mother to daughter cells. However, experimental evidence from *in vitro *studies has shown that DNA methylation states in specific genes may change rapidly in response to environmental stressors. Whether DNA methylation in tumor suppressor genes changes in response to short-term exposure to environmental chemicals is yet to be determined. Tumor suppressor gene hypermethylation is widely proposed to represent one of the very early steps in human carcinogenesis [17]. Even transient DNA methylation changes may reflect condition of cellular stress associated with altered apoptosis, cell cycle control, and cell proliferation that may lead to the accumulation of persistent epigenetic and genetic damage after repeated exposures or in the presence of other pro-carcinogenic insults [18].

Foundry work, a specific condition of exposure to inhalable metal-rich particles, has been associated with increased risk of lung cancer [19]. Even in modern foundry facilities, workers are still exposed to substantially higher levels of airborne PM compared to those found outdoors [10]. In the present study of 63 foundry workers with different levels of usual PM exposure, we examined the association of PM exposure with 4 tumor suppressor genes (i.e., APC, p16, p53 and RASS1FA) promoter DNA methylation levels. To capture the potential effects of short PM exposures, we further evaluated the differences in DNA methylation measured at the beginning and at the end of a work week.

## Material and Methods

### Study population

We recruited 63 male healthy workers free of cancer and cardiovascular and pulmonary disease in an electric steel plant in Brescia, Northern Italy, between April and May 2006 as described previously [20]. All participants had been working in the current job position for at least one year. A self-administered questionnaire was used to collect detailed information on lifestyle, drug use, recent medical conditions, and residential history. Records from the factory administrative and clinical files were used to abstract information on occupational and past medical history. In order to discriminate short- and long-term effects of PM on biomarkers that are relevant to diseases, we obtained blood samples at two different times: i) the baseline sample was collected in the morning of the 1^st ^day of a work week (following two days off work) before the beginning of any work activity; ii) the post-exposure sample was collected at the same time on the 4^th ^work day of the same week. Individual written informed consent and approval from the local Institutional Review Board were obtained before the study.

### Exposure measurement

Measures of the airborne levels of PM mass and metal components obtained in each of the 11 work areas of the plant were used to estimate individual exposures as described before [20]. During the three work days between baseline and post-exposure samples collection, each of the study subjects recorded the time that he spent in each of the work areas. For each of the exposures, we calculated the personal time-weighted average exposure level by multiplying the time spent in each area by the level of PM mass or metal components in the area, which was then divided by the total time spent at work. The exposure levels of each pollutant in each of the work areas have shown very little variability over time, as measures repeated over one year in a subset of subjects showed very high correlation (*r*^2 ^> 0.90). Because all the study subjects reported in the questionnaire have performed their standard work routine during the three days of the study, the time-weighted personal levels of PM mass and metal components, in addition to the exposure during the week of the study, was also a measure of the usual exposure of the study subjects [10].

Measures of PM mass included concentrations of airborne PM with aerodynamic diameters < 10 μm (PM_10_) and < 1 μm (PM_1_) measured using a GRIMM 1100 light-scattering dust analyzer (Grimm Technologies, Inc. Douglasville, GA, USA). We measured air concentrations of individual metal particle components in PM_10_, through multi-elemental analysis performed by means of inductively coupled-plasma mass spectrometer (ICP-MS, ELAN DRC II, Perkin Elmer, Waltham, MA, USA) using the Total Quant method. External calibration was performed using calibration standard 3, stock multi-element (10 μg/ml; Perkin Elmer, Waltham, MA, USA). The coefficient of variations for the metal concentrations obtained in repeated measures varied between 4% and 8%.

### DNA Methylation measurement

We used EDTA tubes to collect 7 ml whole blood that was promptly centrifuged on site at 2500 rpm for 15 minutes. The buffy coat (400 μl) was transferred in a cryovial, immediately frozen in vapour phase of liquid nitrogen, and shipped in nitrogen dry shippers to the laboratory. DNA was extracted using the Wizard Genomic DNA purification kit (Promega, Madison, WI, USA) following the manufacturer's instructions. The samples collected on the 1^st ^and 4^th ^day were processed using the same exact protocols. Promoter regions and amplicons of four promoter CpG sites in APC, p53, or RASSF1A and seven CpG sites in p16 are listed in Additional file 1, **Table S1**. For all assays, we used built-in controls to verify bisulfite conversion efficiency. Compared with other common methods of DNA methylation analysis, pyrosequencing-based assays have the advantage of producing individual measures of methylation at more than one CpG dinucleotide, thus reflecting more accurately DNA methylation in the region.

### Statistical Analysis

For each subject, the pyrosequencing-based analysis of DNA methylation produced eight values each for APC, p53 or RASS1FA (methylation at four CpG dinucleotide sites for each of the genes, replicated in two analytical measurements) and fourteen values for p16 (methylation at seven CpG dinucleotide sites, replicated in two analytical measurements). Each subject was tested twice for baseline and post-exposure DNA sample. We first evaluated differences between baseline and post-exposure samples using the following model: Y_ijk _= β_0 _+ β_1_(Sample)_k _+ β_2_(CpG position)_j _+ ξ_ijk _+ ε_ijk_, where β_0 _is the overall intercept; β_1 _is the regression coefficient for the difference between baseline and post-exposure sample; β_2 _is the regression coefficient for the difference between CpG dinucleotides position; *i = 1,2,...,63 *represents the subject; *j = 1,2...4/7 *represents the CpG dinucleotides position; *k = 1,2 *represents the sample. The random intercept, ξ_ijk_, represents the *i*th individual's deviation from the population mean intercept and models the correlation in the repeated measures within subject. ε_ijk _is the residual error term. We estimated the effect of PM mass and PM metal component on post-exposure DNA sample with the following model: Y_ij _= β_0 _+ β_1_(Exposure)_i _+ β_2_(CpG position)_j _+ ξ_ij _+ ε_ij _where β_0 _is the overall intercept; β_1 _is the regression coefficient representing the exposure effect; β_2 _is the regression coefficient for the difference between CpG dinucleotides position; *i = 1,2,...,63 *represents the subject; *j = 1,2...4/7 *represents the CpG dinucleotides position. The random intercept, ξ_ij_, represents the *i*th individual's deviation from the population mean intercept and models the correlation in the repeated measures within subject. ε_ij _is the residual error term.

Covariates for multivariate models included the following potential confounders: age, body mass index (BMI), smoking and percent granulocytes in the differential blood count. We further conducted analyses without adjusting for percent granulocytes and did not observe meaningful differences in the results from the adjusted models. The independent variables used in multivariable models were selected *a priori *and included general characteristics potentially associated with cancer risk or other carcinogenic exposures. An unstructured covariance structure was used to model the within-subject errors. In order to compare the magnitude of the associations of DNA methylation genes with different exposures, we calculated standardized regression coefficients which express the effects on DNA methylation genes as the fraction of a standard deviation of DNA methylation genes associated with a standard deviation increase in exposure. The Kenword-Roger approximation was used to estimate denominator degrees of freedom. A p-value < 0.05 was considered statistically significant. All statistical analyses were performed in SAS 9.2 (SAS Institute Inc., Cary, NC, USA).

## Results

### Characteristics and exposure levels of the study subjects

Characteristics and exposure levels of the study subjects were reported previously [21]. Briefly, the mean age of the study subjects was 44 years (range between 27 and 55 years). Twenty-five subjects (40%) were current smokers, who reported a mean number of 13.0 (SD = 7.2) cigarettes smoked every day. The mean BMI of the study participants was 26.5 Kg/m^2 ^(SD = 2.7). Table 1 shows the mean estimated levels of inhalable PM mass and air metal concentrations. For both PM mass and metal levels, the study subjects showed wide ranges of exposure levels. For instance, the minimum and maximum PM_10 _levels were 73.72 μg/m^3 ^and 1220.17 μg/m^3^, respectively. For some of the exposures (i.e., aluminum, manganese, zinc, and lead), the maximum individual exposure was more than 200 times higher than the minimum individual exposure.

**Table 1:** Distribution of PM mass and metals contained in PM* among study subjects (N = 63)

Exposure (μg/m^3^)	N	Mean (SD)		Min	Percentile			Max
	
					25^th^	50^th^	75^th^	
*PM_10_*	63	233.42	(214.56)	73.72	152.23	179.45	222.86	1220.17
*PM_1_*	63	8.48	(6.18)	1.71	3.51	9.01	11.35	30.49
*Aluminum*	63	8.50	(18.07)	0.40	1.48	2.05	7.41	84.07
*Manganese*	63	11.26	(30.41)	0.11	1.20	4.63	10.77	174.79
*Nickel*	63	0.30	(0.18)	0.02	0.23	0.25	0.46	0.72
*Zinc*	63	18.85	(26.37)	0.26	1.47	8.45	32.28	129.06
*Arsenic*	63	0.10	(0.1)	0.01	0.02	0.07	0.17	0.31
*Lead*	63	7.53	(17.46)	0.13	0.63	2.87	9.52	99.90
*Iron*	63	32.02	(22.08)	0.96	18.00	25.64	48.69	88.43

*Metals were measured in the PM_10 _fraction of PM mass

The matrix of correlations between exposure levels (Additional file 1, **Table S2) **showed that nickel was positively correlated with all other metals and PM levels (r between 0.22 and 0.84). Zinc also showed positive correlations (r between 0.25 and 0.48) with nickel, arsenic, lead, iron as well as with PM_10 _and PM_1_. Arsenic showed positive correlations (r between 0.25 and 0.84) with manganese, nickel, zinc, lead, and iron. Lead was correlated (r between 0.28 and 0.99) with all other metals. Iron showed positive correlations (r between 0.26 and 0.77) with all other metals, except aluminum, as well as with PM_10 _and PM_1 _levels. PM_10 _and PM_1 _were positively correlated (r = 0.91).

### Changes in DNA methylation during the work week

**Figure 1:**
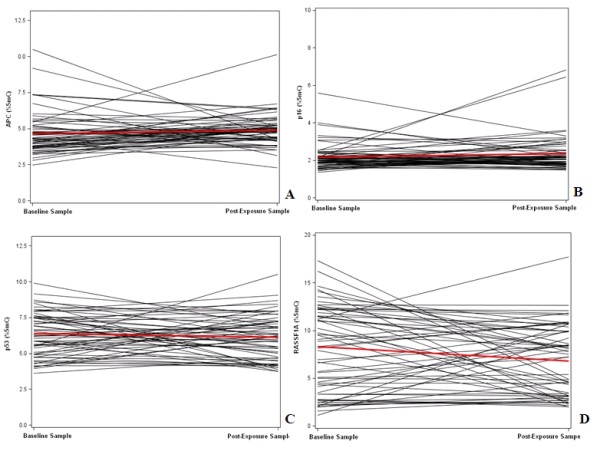
**DNA methylation levels measured in baseline and post-exposure samples**. The spaghetti plots show the changes of individuals' DNA methylation levels in APC (A), P16 (B), P53 (C), RASSF1A (D) (black lines) and the mean change (bolded red lines) between baseline and post-exposure samples.

We compared DNA methylation in the baseline samples with those in the post-exposure samples (Table 2). Individuals trajectories for all subjects are shown in Figure 1. APC methylation showed non-significant increases at all four CpG sites evaluated. When the four CpG sites were combined, the mean APC methylation level significantly increased from baseline 4.65% 5 mC to post-exposure 4.89% 5 mC (p-value = 0.005). DNA methylation levels at the seven individual p16 promoter CpG sites showed inconsistent patterns. Of the seven CpG sites, methylation increased significantly only at CpG site 1 in post-exposure samples (from baseline 2.02% 5 mC to 2.34% 5 mC, p-value = 0.012). The remaining six CpG sites showed non-significant or no increase in post-exposure samples. The mean methylation level of the combined seven CpG sites showed a significant increase from 2.20% 5 mC at baseline to 2.34% 5 mC after 4 work days (post-exposure) (p-value = 0.006). By contrast, p53 methylation decreased significantly at CpG site 4 (from 6.96% 5 mC to 6.59% 5 mC, p-value = 0.006), but not in the other three CpG sites. The mean methylation of the combined four p53 sites showed a significant decrease from 6.36% 5 mC in baseline samples to 6.16% 5 mC in post-exposure samples (p-value = 0.015). RASSF1A methylation showed significant decreases at all four CpG sites evaluated. The mean methylation value of the combined four RASSF1A CpG sites decreased significantly from 8.17% 5 mC in baseline samples to 7.08% 5 mC in post-exposure samples (p-value < 0.001).

**Table 2:** Promoter CpG site DNA methylation of four tumor suppressor genes measured in post-exposure and baseline samples

***CpG site***	*N*	DNA methylation				
	
		Baseline sample		Post-exposure sample		p-value
***APC (%5 mC)***						
*site 1*	60	5.77	(5.41; 6.13)	6.13	(5.77; 6.49)	0.06
*site 2*	60	5.39	(5.10; 5.68)	5.45	(5.16; 5.74)	0.72
*site 3*	60	3.63	(3.32; 3.93)	3.92	(3.62; 4.23)	0.11
*site 4*	60	3.86	(3.56; 4.15)	4.06	(3.77; 4.35)	0.25
*All sites combined*	60	4.65	(3.70; 5.61)	4.89	(3.94; 5.85)	**0.005**

***p16 (%5 mC)***						
*site 1*	61	2.02	(1.81; 2.22)	2.34	(2.13; 2.55)	**0.012**
*site 2*	61	2.75	(2.51; 2.98)	2.9	(2.66; 3.14)	0.20
*site 3*	61	1.66	(1.43; 1.89)	1.88	(1.65; 2.11)	0.11
*site 4*	61	2.21	(2.05; 2.36)	2.17	(2.01; 2.33)	0.70
*Site5*	61	1.84	(1.57; 2.12)	1.93	(1.65; 2.20)	0.60
*Site 6*	61	1.71	(1.49; 1.92)	1.78	(1.56; 2.00)	0.58
*Site 7*	61	3.11	(2.83; 3.40)	3.31	(3.03; 3.60)	0.15
*All sites combined*	61	2.2	(1.78; 2.62)	2.34	(1.92; 2.76)	**0.006**

***p53 (%5 mC)***						
*site 1*	58	3.02	(2.83; 3.22)	2.93	(2.73; 3.13)	0.16
*site 2*	58	11.88	(11.26; 12.50)	11.56	(10.94; 12.18)	0.13
*site 3*	58	3.57	(3.37; 3.78)	3.56	(3.35; 3.76)	0.85
*site 4*	58	6.96	(6.52; 7.41)	6.59	(6.14; 7.03)	**0.006**
*All sites combined*	58	6.36	(2.93; 9.79)	6.16	(2.73; 9.60)	**0.015**

***RASSF1A (%5 mC)***						
*site 1*	57	3.68	(3.23; 4.13)	3.14	(2.69; 3.59)	**0.003**
*site 2*	57	10.14	(9.01; 11.27)	8.59	(7.45; 9.72)	**< 0.001**
*site 3*	57	8.43	(7.16; 9.70)	7.21	(5.94; 8.49)	**0.009**
*site 4*	57	10.61	(9.34; 11.88)	9.22	(7.95; 10.49)	**0.002**
*All sites combined*	57	8.17	(5.44; 10.90)	7.08	(4.36; 9.81)	**< 0.001**

### Associations of DNA methylation with exposure to PM mass and metal component

The correlations of PM mass and its metal components with post-exposure promoter DNA methylation were examined. This set of analyses takes advantage of the large differences in exposure levels among the study subjects. We present results from unadjusted models as well as from models adjusted for age, BMI, smoking, proportion of granulocytes in the differential blood count. For each gene, the mean methylation levels of all CpG sites (Table 3) and the methylation levels of each individual CpG site (Additional file 1, **Table S3, S4, S5, and S6**) were regressed over the exposure levels. The mean methylation level of four CpG sites in APC promoter region was positively associated with the levels of PM_10 _(β_std _= 0.27, 95% CI: 0.14-0.40), PM_1 _(β_std _= 0.23, 95% CI: 0.10-0.37) and aluminum (β_std _= 0.15, 95% CI: 0.00-0.29) in the unadjusted regression. The adjusted regression confirmed significant associations of APC methylation with PM_10 _(β_std _= 0.27, 95% CI: 0.13-0.40), and PM_1 _(β_std _= 0.23, 95% CI: 0.09-0.38), and a borderline significant association with aluminum (β_std _= 0.14, 95% CI: 0.00-0.29). The mean methylation level of seven CpG sites in p16 promoter region was positively associated with the levels of zinc in both the unadjusted (β_std _= 0.18, 95% CI 0.00-0.35) and adjusted (β_std _= 0.19, 95% CI: 0.02-0.37) regressions. RASSF1A promoter methylation level measured in four CpG sites was negatively associated with the level of zinc exposure in both the unadjusted (β_std _= -0.18, 95% CI: -0.35--0.01) and adjusted regressions (β_std _= -0.21, 95% CI: -0.37--0.05). p53 methylation did not show any significant associations with the exposures evaluated.

**Table 3:** Association of PM mass and metal components with methylation of four tumor suppressor genes measured in post-exposure samples (N = 63)

Exposures	APC		p16		p53		RASSF1A	
	
	β_std_	95% CI	β_std_	95% CI	β_std_	95% CI	β_std_	95% CI
**Unadjusted regression**								
PM_10_	0.27^a^	0.14 to 0.40	0.02	-0.17 to 0.20	0.00	-0.12 to 0.11	0.01	-0.16 to 0.18
PM_1_	0.23^b^	0.10 to 0.37	0.02	-0.16 to 0.20	-0.06	-0.17 to 0.04	-0.01	-0.18 to 0.16
Aluminium	0.15^c^	0.00 to 0.29	0.00	-0.18 to 0.18	0.04	-0.07 to 0.15	0.01	-0.15 to 0.18
Manganese	0.11	-0.03 to 0.26	-0.03	-0.22 to 0.15	-0.02	-0.15 to 0.11	0.11	-0.05 to 0.28
Nickel	0.08	-0.07 to 0.23	0.00	-0.18 to 0.18	0.04	-0.06 to 0.14	-0.02	-0.19 to 0.15
Zinc	0.06	-0.08 to 0.21	0.18^c^	0.00 to 0.35	-0.06	-0.15 to 0.03	-0.18^c^	-0.35 to -0.01
Arsenic	0.01	-0.15 to 0.17	-0.02	-0.20 to 0.16	0.05	-0.05 to 0.15	0.01	-0.17 to 0.18
Lead	0.12	-0.03 to 0.26	-0.01	-0.19 to 0.17	-0.04	-0.17 to 0.09	0.08	-0.09 to 0.25
Iron	0.05	-0.10 to 0.20	-0.02	-0.20 to 0.16	-0.03	-0.12 to 0.07	-0.06	-0.24 to 0.12
								
Multivariable regression*								
PM_10_	0.27^a^	0.13 to 0.40	0.01	-0.17 to 0.20	0.00	-0.12 to 0.12	0.03	-0.13 to 0.19
PM_1_	0.23^b^	0.09 to 0.38	0.02	-0.17 to 0.21	-0.06	-0.16 to 0.05	0.04	-0.14 to 0.21
Aluminium	0.14	0.00 to 0.29	0.00	-0.19 to 0.18	0.05	-0.06 to 0.17	0.05	-0.11 to 0.21
Manganese	0.12	-0.03 to 0.26	-0.03	-0.22 to 0.15	-0.02	-0.15 to 0.12	0.12	-0.04 to 0.28
Nickel	0.08	-0.08 to 0.23	0.00	-0.18 to 0.19	0.04	-0.06 to 0.13	-0.03	-0.19 to 0.13
Zinc	0.08	-0.07 to 0.23	0.19^c^	0.02 to 0.37	-0.07	-0.16 to 0.03	-0.21^c^	-0.37 to -0.05
Arsenic	0.02	-0.14 to 0.18	-0.01	-0.20 to 0.18	0.04	-0.07 to 0.14	-0.04	-0.21 to 0.13
Lead	0.12	-0.02 to 0.27	-0.01	-0.19 to 0.18	-0.05	-0.18 to 0.09	0.08	-0.08 to 0.24
Iron	0.05	-0.10 to 0.21	-0.01	-0.20 to 0.17	-0.04	-0.13 to 0.06	-0.08	-0.25 to 0.09

* multivariable mixed effects models adjusted for age, BMI, smoking, % of granulocytes

^a ^p ≤ 0.0001; ^b ^p ≤ 0.001; ^c ^p ≤ 0.05

### Correlation with other biomarkers previously examined in the study

In addition, we have previously examined a number of biomarkers in this study population, including mitochondria DNA copy number variation, global methylation levels measured in two repetitive elements, Alu and LINE-1, and hTERT, a telomere length maintenance gene [10,22,23]. We did not observe significant correlations between these markers with DNA methylation level in 4 tumor suppressor genes (Additional file 1, **Table S7**).

## Discussion

In the present study, we showed that the mean methylation levels of promoter CpG sites were significantly associated with the exposure to PM and certain metal components. In particular, the mean DNA methylation levels of the CpG sites measured in APC or p16 promoter increased significantly in post-exposure DNA samples. By contrast, the mean of p53 or RASSF1A promoter CpG sites methylation decreased in post-exposure DNA samples. CpG site-specific analyses did not show consistent patterns of DNA methylation changes in any of the four genes.

Evidence in human subjects is rapidly mounting to establish associations of DNA methylation alterations with environmental exposures. Such methylation changes can persist over time even in the absence of the conditions that established them and even accumulate in response to continuous exposure [24-26]. Exposure to air pollution, particularly to ambient PM, has been associated with increased lung cancer risk [1,2]. Aberrant DNA promoter methylation of tumor suppresser genes, including hypermethylation of p16 [27,28], APC [29] and RASSF1A [28,30], and hypomethylation of p53 [31], has been observed in blood leukocyte DNA from lung cancer patients, suggesting that PBL DNA methylation may serve as a cancer-related biomarker.

The four genes of interest in our study are involved in cell-cycle control (p16), invasion and metastasis (APC), apoptosis (p53) and Ras signaling (RASSF1A) and have been shown to be altered in lung cancer patients' blood DNA [27-31]. Some of our findings are in line with previous reports. Zhang *et al *and Chanda *et al *found hypermethylation of p16 tumor suppressor gene in blood DNA from individuals exposed to emissions from indoor unventilated-stove coal usage [9] and individuals exposed to high-level arsenic [8]. Expression of p16, one of the most promising early epigenetic markers for the detection of lung cancer [32], was demonstrated to be regulated by promoter methylation [33]. Hypermethylation of p16 has been detected in blood leukocyte DNA from cancer-free smokers [34], lung cancer smokers [27,28] and in lung tissue from diesel exhaust-exposed rats [35]. APC promoter hypermethylation, another early event of tumorigenesis, has been extensively studied in lung tumors [36]. APC promoter hypermethylation has also been seen in lung tissue in healthy subjects exposed to cigarette smoke [37] and blood in lung cancer patients [29]. Our finding, for the first time, shows that air pollution may induce blood leukocyte APC promoter hypermethylation. p53 is a key factor in DNA damage-signaling pathway, and p53 hypomethylation is associated with DNA double strand breaks and chromosomal instability [31]. In a previous human study, we observed p53 hypomethylation in blood DNA in Polish male non-smoking coke-oven workers exposed to polycyclic aromatic hydrocarbons (PAHs) [38,39], which is similar to findings in smoker lung cancer patients [31]. Our finding further suggests that p53 blood leukocyte hypomethylation may also occur in healthy subjects who are exposed to PM. Although hypermethylated RASSF1A promoter is frequently observed in the blood DNA of lung cancer patients [28,30], we found an inverse association between mean promoter RASS1FA methylation and PM exposure. It is possible that our results might represent tissue-specific effects of PM exposures on blood leukocytes, as well as a false positive finding due to the limited sample size. Different tumor suppressor genes may behave differently with respect to carcinogens. Air pollution may cause hypo- or hypermethylation in each individual gene depending on the role of the gene in cancer development. In our study, we observed positive association of p16 methylation with zinc, and negative association of RASSF1A methylation with zinc in both unadjusted and adjusted regressions. Zinc is a major component in PM in steel-production plants, and was present at high levels in PM measured in the present study (Table 1). Zinc inhalation has been shown to induce inflammation and oxidative stress in animal studies [40,41]. Kodavanti *et al *have also demonstrated that leachable zinc from PM induced both pulmonary and systemic changes in multiple *in vivo *toxicology experiments [42]. Furthermore, a study has shown that inhalation of soluble zinc sulfate, even at low levels (10 μg/m^3^), caused gene expression change in heart tissue in healthy rats [43].

PM and its metal components may affect DNA methylation through several cellular processes, including oxidative stress/reactive oxygen species (ROS) generation and systemic inflammation/immune deficiency [44,45], the two major components in the etiology of cancer [46]. These cellular processes have been shown to be induced by exposure to PM [47,48] and associated with altered DNA methylation patterns [49].

Our study was based on occupationally PM-exposed volunteers who worked in several work areas of the same factory and did not include a different population of subjects without a specific condition of exposure to PM. Limiting our investigation to individuals who have all been working in the same work facility avoided potential concerns related to the selection of external referents who might have differed from the exposed population in terms of socioeconomic factors and other characteristics determining hiring into the plant [50]. The differences in the individual levels of exposure in our study group were large, which provided sufficient contrast for identifying exposure-related changes in DNA methylation. For example, the lowest level of PM_10 _observed in our study population (73.72 μg/m^3^) was only marginally higher than ambient PM_10 _levels measured in the geographic area where the plant is located [average annual ambient PM_10 _levels between 41 and 57 μg/m^3 ^were recorded in the year of the study by different ambient monitoring stations in Brescia area] [51], whereas the highest level was 1220.17 μg/m^3^. It is worth noting that, although our study was based on a group of foundry workers with higher average exposures than the general population, the levels of exposure to metals in our study were all lower than the commonly accepted threshold limits for industrial settings [52]. As foundry workers may have additional exposures [53-57], we cannot exclude that exposures other than PM might have contributed to the observed effects, although study subjects in our study were in a modern facility with state-of-the-art systems for exposure reduction. A limitation of the study is that it did not include an unexposed comparison group. We cannot exclude that effects of potential changes in life-style from the start to the end of the week might have played a role in generating the observed associations. However, this group of foundry workers showed wide differences in exposures between individuals. The dose-response associations that we found between PM and APC methylation, and between zinc and p16 and RASSF1A methylation all hint to specific exposure-related effects.

In summary, we observed hypermethylation of p16 and APC, and hypomethylation of RASSF1A and p53 in a group of healthy foundry workers who had higher levels of exposure to PM compared to the general populations. Altered DNA methylation of tumor suppressor genes in easily obtainable cells such as PBLs may have potentials for developing biomarkers to detect biological alterations in PM-exposed subjects. These results suggest that such methylation alterations may reflect processes related to PM-induced lung carcinogenesis. However, because our study did not include lung cancer or other carcinogenesis-related endpoints, we cannot make inference on the biological significance of our findings. Further studies in larger populations are required before any firm conclusion could be reached on whether PM exposed individuals with such DNA methylation alterations are at higher risk for lung cancer.

## Competing interests

The authors declare that they have no competing interests.

## Authors' contributions

LH and AB generated the study concept, directed statistical analysis, and drafted manuscript. ZX contributed to data interpretation and manuscript writing. LA and FN conducted the statistical analysis, prepared the results in tabular form and contributed to the data interpretation. LT and LC designed the methylation assays and performed the DNA methylation analysis. GR prepared the protocols for and coordinated the acquisition, preprocessing and handling of the biospecimens, and implemented QC/QA procedures. MB, BM, PA, and PAB planned and directed the study subject's recruitment and exposure assessment. All authors read and approved the final manuscript.

## Supplementary Material

Additional file 1 **Supplementary Table S1, S2, S3, S4, S5, S6 and S7**. This file contains supplementary Tables S1, S2, S3, S4, S5, S6 and S7. Click here for file
