# Supplementary material for: Ambient PM exposure and DNA methylation in tumor suppressor genes: a cross-sectional study
Source: Part Fibre Toxicol. 2011 Aug 30;8:25. doi: 10.1186/1743-8977-8-25 (PMC3180673; doi:10.1186/1743-8977-8-25)
Supplement: Additional file 1 — Supplementary Table S1, S2, S3, S4, S5, S6 and S7. This file contains supplementary Tables S1, S2, S3, S4, S5, S6 and S7. [file 1743-8977-8-25-S1.PDF]

Supplementary Table S1. Promoters, amplicons, and CpG sites for four tumor suppressor genes

Gene	Chromosome	Promoter		Amplicon		CpG site
		Start	***End***	***Start***	***End***	
**APC**	5	112100983	112101583	112101225	112101322	site 1: 112101248; site 2: 112101250; site 3: 112101264; site 4: 112101273
**P53**	17	7531143	7531743	7531409	7531628	site 1: 7531486; site 2: 7531473; site 3: 7531469; site 4: 7531458
**P16**	9	21964701	21965538	21965321	21965395	site 1: 21965350; site 2: 21965355; site 3: 21965357; site 4:21965361; site 5: 21965365; site 6: 21965368; site 7: 21965368
**RASSF1A**	3	50353270	50353877	50353497	50353694	site 1: 50353604; site 2: 50353614; site 3: 50353616; site 4: 50353628

Supplementary Table S2. Pearson's correlation coefficient between pairs of pollutants

		PM_{10}	PM_1	Aluminum	Manganese	Nickel	Zinc	Arsenic	Lead	Iron	Chromium
**PM_{10}**		1									
**PM_1**	*r*	0.90	1								
	p-value	*<0.001*									
**Aluminum**	*r*	0.81	0.63	1							
	p-value	*<0.001*	*<0.001*								
**Manganese**	*r*	0.82	0.60	0.75	1						
	p-value	*<0.001*	*<0.001*	*<0.001*							
**Nickel**	*r*	0.35	0.22	0.46	0.39	1					
	p-value	*0.006*	*0.090*	*<0.001*	*0.002*						
**Zinc**	*r*	0.34	0.28	0.18	0.21	0.32	1				
	p-value	*0.007*	*0.028*	*0.163*	*0.103*	*0.011*					
**Arsenic**	*r*	0.04	-0.20	0.15	0.31	0.84	0.25	1			
	p-value	*0.745*	*0.119*	*0.238*	*0.014*	*<0.001*	*0.048*				
**Lead**	*r*	0.86	0.65	0.75	0.99	0.38	0.35	0.28	1		
	p-value	*<0.001*	*<0.001*	*<0.001*	*<0.001*	*0.002*	*0.005*	*0.026*			
**Iron**	*r*	0.27	0.26	0.15	0.28	0.77	0.48	0.70	0.32	1	
	p-value	*0.029*	*0.041*	*0.245*	*0.026*	*<0.001*	*<0.001*	*<0.001*	*0.011*		

Supplementary Table S3. Association of PM mass and metal components with methylation of specific promoter CpG sites in APC measured in post-exposure samples (N=63)

	Post-exposure sample			
	β_{std}	95% CI	β^*_{std}	95% CI*
**PM_{10}**				
site 1	0.50	0.28 to 0.73	0.51	0.27 to 0.76
site 2	0.45	0.22 to 0.68	0.50	0.24 to 0.75
site 3	0.19	-0.06 to 0.44	0.20	-0.09 to 0.48
site 4	0.42	0.19 to 0.65	0.39	0.14 to 0.64
**PM_1**				
site 1	0.43	0.2 to 0.66	0.43	0.17 to 0.69
site 2	0.40	0.16 to 0.63	0.42	0.16 to 0.69
site 3	0.16	-0.09 to 0.42	0.15	-0.14 to 0.44
site 4	0.39	0.15 to 0.62	0.33	0.07 to 0.58
**Aluminum**				
site 1	0.29	0.04 to 0.53	0.29	0.02 to 0.55
site 2	0.24	-0.01 to 0.49	0.27	0.00 to 0.54
site 3	0.06	-0.19 to 0.32	0.06	-0.22 to 0.34
site 4	0.28	0.03 to 0.53	0.25	0.00 to 0.51
**Manganese**				
site 1	0.29	0.05 to 0.54	0.28	0.00 to 0.56
site 2	0.18	-0.08 to 0.43	0.18	-0.11 to 0.47
site 3	-0.04	-0.3 to 0.21	-0.06	-0.36 to 0.23
site 4	0.23	-0.02 to 0.48	0.21	-0.05 to 0.48
**Nickel**				
site 1	0.12	-0.14 to 0.38	0.14	-0.14 to 0.42
site 2	0.17	-0.09 to 0.43	0.21	-0.06 to 0.49
site 3	0.03	-0.23 to 0.29	0.05	-0.23 to 0.34
site 4	0.15	-0.11 to 0.41	0.16	-0.10 to 0.42
**Zinc**				
site 1	0.16	-0.09 to 0.42	0.20	-0.07 to 0.47
site 2	0.21	-0.04 to 0.46	0.23	-0.04 to 0.5
site 3	0.02	-0.23 to 0.28	0.07	-0.21 to 0.35
site 4	-0.04	-0.30 to 0.22	0.03	-0.22 to 0.29
**Arsenic**				
site 1	0.02	-0.25 to 0.29	0.03	-0.26 to 0.33
site 2	0.05	-0.23 to 0.32	0.07	-0.23 to 0.37
site 3	-0.02	-0.29 to 0.26	0.02	-0.28 to 0.32
site 4	0.02	-0.25 to 0.29	0.07	-0.20 to 0.35
**Lead**				
site 1	0.31	0.06 to 0.55	0.30	0.02 to 0.57
site 2	0.20	-0.05 to 0.45	0.21	-0.08 to 0.49
site 3	-0.03	-0.29 to 0.22	-0.05	-0.34 to 0.24
site 4	0.22	-0.03 to 0.47	0.21	-0.06 to 0.47
**Iron**				
site 1	0.09	-0.17 to 0.35	0.10	-0.18 to 0.38
site 2	0.11	-0.15 to 0.37	0.14	-0.14 to 0.42
site 3	-0.01	-0.27 to 0.25	0.00	-0.29 to 0.28
site 4	0.12	-0.14 to 0.38	0.12	-0.14 to 0.38

* Multivariable regression models adjusted for age, BMI, smoking, % of granulocytes

Supplementary Table S4. Association of PM mass and metal components with methylation of specific promoter CpG sites in p16 measured in post-exposure samples (N=63)

	Post-exposure sample			
	β_{std}	95% CI	β^*_{std}	95% CI*
**PM_{10}**				
site 1	0.13	-0.12 to 0.38	0.14	-0.13 to 0.40
site 2	0.09	-0.16 to 0.35	0.05	-0.22 to 0.33
site 3	-0.03	-0.29 to 0.22	0.00	-0.25 to 0.25
site 4	-0.14	-0.40 to 0.11	-0.17	-0.43 to 0.10
site.5	0.02	-0.23 to 0.28	0.07	-0.18 to 0.33
site.6	-0.04	-0.30 to 0.22	-0.10	-0.38 to 0.185
site.7	-0.02	-0.27 to 0.24	-0.01	-0.29 to 0.26
**PM_1**				
site 1	0.16	-0.1 to 0.41	0.15	-0.12 to 0.41
site 2	0.08	-0.18 to 0.33	0.05	-0.22 to 0.33
site 3	-0.01	-0.27 to 0.24	0.02	-0.23 to 0.26
site 4	-0.13	-0.38 to 0.13	-0.14	-0.41 to 0.12
site.5	0.04	-0.22 to 0.29	0.08	-0.18 to 0.33
site.6	-0.01	-0.27 to 0.25	-0.08	-0.36 to 0.20
site.7	-0.03	-0.29 to 0.23	-0.05	-0.32 to 0.23
**Aluminum**				
site 1	0.08	-0.17 to 0.34	0.09	-0.17 to 0.34
site 2	0.08	-0.17 to 0.34	0.02	-0.25 to 0.28
site 3	-0.06	-0.32 to 0.19	-0.03	-0.28 to 0.21
site 4	-0.07	-0.33 to 0.18	-0.08	-0.34 to 0.18
site.5	-0.01	-0.26 to 0.25	0.02	-0.23 to 0.27
site.6	-0.06	-0.31 to 0.20	-0.09	-0.36 to 0.18
site.7	0.01	-0.25 to 0.26	0.01	-0.26 to 0.28
**Manganese**				
site 1	0.04	-0.22 to 0.30	0.05	-0.22 to 0.32
site 2	0.02	-0.24 to 0.27	-0.05	-0.33 to 0.22
site 3	-0.05	-0.3 to 0.21	-0.01	-0.26 to 0.24
site 4	-0.16	-0.42 to 0.09	-0.20	-0.47 to 0.06
site.5	-0.03	-0.29 to 0.22	0.01	-0.25 to 0.26
site.6	-0.09	-0.35 to 0.16	-0.15	-0.43 to 0.13
site.7	-0.08	-0.33 to 0.18	-0.07	-0.35 to 0.20
**Nickel**				
site 1	-0.05	-0.31 to 0.21	-0.05	-0.31 to 0.21
site 2	0.01	-0.25 to 0.27	-0.03	-0.3 to 0.23
site 3	0.00	-0.26 to 0.26	0.05	-0.20 to 0.29
site 4	-0.06	-0.32 to 0.20	-0.10	-0.35 to 0.16
site.5	0.09	-0.17 to 0.34	0.11	-0.14 to 0.35
site.6	-0.08	-0.34 to 0.18	-0.12	-0.39 to 0.15
site.7	0.04	-0.22 to 0.30	0.02	-0.24 to 0.29
**Zinc**				
site 1	0.21	-0.04 to 0.46	0.28	0.04 to 0.53
site 2	0.35	0.11 to 0.59	0.38	0.14 to 0.62
site 3	0.12	-0.13 to 0.38	0.21	-0.02 to 0.45
site 4	-0.01	-0.27 to 0.25	0.03	-0.23 to 0.29

site.5	0.31	0.07 to 0.56	0.35	0.13 to 0.58
site.6	0.07	-0.19 to 0.32	0.05	-0.22 to 0.32
site.7	0.12	-0.14 to 0.37	0.10	-0.16 to 0.36
**Arsenic**				
site 1	-0.10	-0.36 to 0.16	-0.10	-0.36 to 0.17
site 2	-0.04	-0.3 to 0.22	-0.09	-0.36 to 0.19
site 3	0.01	-0.25 to 0.27	0.04	-0.21 to 0.28
site 4	-0.09	-0.35 to 0.17	-0.13	-0.4 0 to 0.13
site.5	0.06	-0.20 to 0.33	0.06	-0.19 to 0.32
site.6	-0.12	-0.38 to 0.15	-0.14	-0.42 to 0.14
site.7	0.06	-0.20 to 0.33	0.06	-0.21 to 0.34
**Lead**				
site 1	0.08	-0.18 to 0.33	0.11	-0.16 to 0.37
site 2	0.07	-0.18 to 0.33	0.02	-0.25 to 0.29
site 3	-0.03	-0.29 to 0.23	0.03	-0.22 to 0.28
site 4	-0.16	-0.41 to 0.09	-0.19	-0.45 to 0.08
site.5	0.01	-0.25 to 0.26	0.07	-0.19 to 0.32
site.6	-0.08	-0.33 to 0.18	-0.13	-0.41 to 0.15
site.7	-0.06	-0.31 to 0.20	-0.05	-0.33 to 0.22
**Iron**				
site 1	-0.05	-0.31 to 0.21	-0.04	-0.30 to 0.21
site 2	-0.01	-0.27 to 0.25	-0.02	-0.28 to 0.24
site 3	0.03	-0.23 to 0.29	0.08	-0.16 to 0.32
site 4	-0.19	-0.44 to 0.06	-0.22	-0.47 to 0.03
site.5	0.10	-0.15 to 0.36	0.14	-0.10 to 0.38
site.6	-0.15	-0.41 to 0.10	-0.19	-0.45 to 0.08
site.7	-0.02	-0.28 to 0.24	-0.02	-0.28 to 0.24

* Multivariable regression models adjusted for age, BMI, smoking, % of granulocytes

Supplementary Table S5. Association of PM mass and metal components with methylation of specific promoter CpG sites in p53 measured in post-exposure samples (N=63)

	Post-exposure sample			
	β_{std}	95% CI	β^*_{std}	95% CI*
**PM_{10}**				
site 1	0.00	-0.31 to 0.32	-0.01	-0.35 to 0.33
site 2	0.07	-0.25 to 0.39	0.04	-0.30 to 0.38
site 3	0.03	-0.29 to 0.35	0.00	-0.35 to 0.34
site 4	-0.16	-0.48 to 0.15	-0.21	-0.55 to 0.13
**PM_1**				
site 1	-0.10	-0.39 to 0.19	-0.13	-0.44 to 0.18
site 2	-0.06	-0.35 to 0.23	-0.05	-0.36 to 0.27
site 3	-0.14	-0.43 to 0.15	-0.19	-0.50 to 0.12
site 4	-0.28	-0.56 to 0.00	-0.30	-0.61 to 0.00
**Aluminum**				
site 1	0.24	-0.06 to 0.54	0.22	-0.09 to 0.54
site 2	0.05	-0.25 to 0.35	0.09	-0.23 to 0.41
site 3	0.18	-0.12 to 0.48	0.22	-0.10 to 0.54
site 4	0.06	-0.25 to 0.36	0.06	-0.26 to 0.39
**Manganese**				
site 1	-0.16	-0.51 to 0.19	-0.17	-0.55 to 0.21
site 2	0.04	-0.32 to 0.39	-0.06	-0.44 to 0.33
site 3	0.02	-0.34 to 0.37	0.02	-0.37 to 0.40
site 4	-0.15	-0.5 to 0.20	-0.22	-0.61 to 0.16
**Nickel**				
site 1	0.12	-0.14 to 0.39	0.17	-0.10 to 0.45
site 2	0.06	-0.21 to 0.33	0.06	-0.22 to 0.34
site 3	0.29	0.03 to 0.55	0.28	0.01 to 0.54
site 4	0.03	-0.24 to 0.30	0.05	-0.24 to 0.33
**Zinc**				
site 1	-0.19	-0.44 to 0.06	-0.14	-0.41 to 0.13
site 2	-0.09	-0.35 to 0.17	-0.12	-0.39 to 0.15
site 3	-0.03	-0.29 to 0.22	-0.02	-0.3 to 0.25
site 4	-0.24	-0.49 to 0.01	-0.23	-0.5 to 0.04
**Arsenic**				
site 1	0.04	-0.24 to 0.31	0.11	-0.18 to 0.40
site 2	0.09	-0.18 to 0.36	0.03	-0.26 to 0.32
site 3	0.30	0.03 to 0.56	0.30	0.02 to 0.58
site 4	0.08	-0.20 to 0.35	0.08	-0.22 to 0.37
**Lead**				
site 1	-0.21	-0.56 to 0.13	-0.22	-0.59 to 0.15
site 2	-0.01	-0.36 to 0.34	-0.10	-0.47 to 0.28
site 3	-0.02	-0.37 to 0.33	-0.03	-0.40 to 0.35
site 4	-0.23	-0.57 to 0.12	-0.30	-0.67 to 0.07
**Iron**				
site 1	-0.09	-0.35 to 0.17	-0.05	-0.32 to 0.23
site 2	-0.06	-0.32 to 0.20	-0.09	-0.36 to 0.19
site 3	0.13	-0.13 to 0.39	0.09	-0.19 to 0.36
site 4	-0.15	-0.41 to 0.11	-0.16	-0.44 to 0.12

* Multivariable regression models adjusted for age, BMI, smoking, % of granulocytes

Supplementary Table S6. Association of PM mass and metal components with methylation of specific promoter CpG sites in RASSF1A measured in post-exposure samples (N=63)

	Post-exposure sample			
	β_{std}	**95% CI**	β^*_{std}	**95% CI***
**PM_{10}**				
site 1	0.21	-0.04 to 0.47	0.21	0.07 to 0.48
site 2	-0.02	-0.28 to 0.24	-0.03	-0.31 to 0.25
site 3	0.00	-0.26 to 0.25	0.01	-0.27 to 0.30
site 4	0.00	-0.26 to 0.24	0.00	-0.28 to 0.29
**PM_1**				
site 1	0.13	-0.13 to 0.39	0.16	-0.12 to 0.44
site 2	-0.05	-0.31 to 0.21	-0.03	-0.32 to 0.26
site 3	-0.01	-0.28 to 0.25	0.03	-0.26 to 0.32
site 4	-0.03	-0.30 to 0.23	0.02	-0.27 to 0.31
**Aluminum**				
site 1	0.17	-0.08 to 0.42	0.20	-0.07 to 0.46
site 2	0.00	-0.26 to 0.25	0.05	-0.22 to 0.32
site 3	0.00	-0.26 to 0.24	0.06	-0.21 to 0.33
site 4	0.02	-0.24 to 0.28	0.06	-0.21 to 0.34
**Manganese**				
site 1	0.30	0.06 to 0.55	0.29	0.03 to 0.56
site 2	0.14	-0.12 to 0.39	0.15	-0.12 to 0.43
site 3	0.14	-0.11 to 0.40	0.17	-0.10 to 0.45
site 4	0.16	-0.10 to 0.41	0.15	-0.13 to 0.43
**Nickel**				
site 1	0.05	-0.21 to 0.31	0.08	-0.18 to 0.35
site 2	-0.03	-0.29 to 0.23	-0.01	-0.28 to 0.26
site 3	-0.02	-0.28 to 0.24	-0.01	0.28 to 0.26
site 4	-0.07	-0.33 to 0.19	-0.07	-0.34 to 0.20
**Zinc**				
site 1	-0.20	-0.46 to 0.06	-0.21	-0.47 to 0.06
site 2	-0.24	-0.50 to 0.02	-0.27	-0.53 to 0.00
site 3	-0.24	-0.50 to 0.02	-0.28	-0.54 to -0.02
site 4	-0.29	-0.54 to -0.04	-0.35	-0.61 to -0.09
**Arsenic**				
site 1	0.06	-0.20 to 0.32	0.04	-0.24 to 0.32
site 2	0.02	-0.25 to 0.28	-0.01	-0.29 to 0.27
site 3	0.02	-0.24 to 0.29	-0.03	-0.31 to 0.25
site 4	-0.02	-0.29 to 0.24	-0.10	-0.38 to 0.18
**Lead**				
site 1	0.25	0.01 to 0.50	0.24	-0.03 to 0.51
site 2	0.09	-0.17 to 0.35	0.10	-0.18 to 0.38
site 3	0.10	-0.16 to 0.35	0.12	-0.16 to 0.39
site 4	0.10	-0.15 to 0.36	0.09	-0.19 to 0.37
**Iron**				
site 1	-0.02	-0.30 to 0.25	-0.02	-0.30 to 0.26
site 2	-0.09	-0.36 to 0.18	-0.10	-0.38 to 0.17
site 3	-0.06	-0.33 to 0.21	-0.09	0.37 to 0.19
site 4	-0.14	-0.41 to 0.13	-0.16	-0.44 to 0.11

* Multivariable regression models adjusted for age, BMI, smoking, % of granulocytes

Supplementary Table S7. Pearson's Correlation Coefficient between the four tumor suppressor genes and other biomarkers in the same population

	**MtDNAcn**		**iNOS**		**Alu**		**LINE-1**		**hTERT**	
	r	*p-value*	*r*	*p-value*	*r*	*p-value*	*r*	*p-values*	*r*	*p-value*
APC	0.16	0.22	-0.001	0.99	-0.08	0.52	0.05	0.70	-0.21	0.12
P16	-0.09	0.48	0.003	0.98	-0.21	0.11	-0.15	0.25	-0.28	0.03
p53	0.13	0.32	-0.07	0.59	-0.08	0.55	0.18	0.17	0.05	0.71
RASSF1A	0.07	0.58	-0.11	0.39	0.13	0.31	0.15	0.26	0.22	0.10

MtDNAcn, mitochondrial DNA copy number; iNOS, inducible nitric oxide synthase; LINE-1, long interspersed nuclear element-1; hTERT, human telomerase reverse transcriptase
